# Perilla Seed Oil Alleviates Gut Dysbiosis, Intestinal Inflammation and Metabolic Disturbance in Obese-Insulin-Resistant Rats

**DOI:** 10.3390/nu13093141

**Published:** 2021-09-09

**Authors:** Napapan Kangwan, Wasana Pratchayasakul, Aphisek Kongkaew, Komsak Pintha, Nipon Chattipakorn, Siriporn C. Chattipakorn

**Affiliations:** 1 Division of Physiology, School of Medical Sciences, University of Phayao, Phayao 56000, Thailand; napapan.kangwan@gmail.com; 2 Neurophysiology Unit, Cardiac Electrophysiology Research and Training Center, Faculty of Medicine, Chiang Mai University, Chiang Mai 50200, Thailand; wpratchayasakul@gmail.com (W.P.); nchattip@gmail.com (N.C.); 3 Cardiac Electrophysiology Unit, Department of Physiology, Faculty of Medicine, Chiang Mai University, Chiang Mai 50200, Thailand; 4 Center of Excellence in Cardiac Electrophysiology Research, Chiang Mai University, Chiang Mai 50200, Thailand; 5 Research Administration Section, Faculty of Medicine, Chiang Mai University, Chiang Mai 50200, Thailand; moromo046@gmail.com; 6 Department of Biochemistry, School of Medical Sciences, University of Phayao, Phayao 56000, Thailand; komsakjo@gmail.com; 7 Department of Oral Biology and Diagnostic Sciences, Faculty of Dentistry, Chiang Mai University, Chiang Mai 50200, Thailand

**Keywords:** gut microbiota, omega-3 fatty acids, inflammation, insulin resistance, systemic inflammation

## Abstract

Background: High-fat diet (HFD) consumption induced gut dysbiosis, inflammation, obese-insulin resistance. Perilla seed oil (PSO) is a rich source of omega-3 polyunsaturated fatty acids with health promotional effects. However, the effects of PSO on gut microbiota/inflammation and metabolic disturbance in HFD-induced obesity have not been investigated. Therefore, we aimed to compare the effects of different doses of PSO and metformin on gut microbiota/inflammation, and metabolic parameters in HFD-fed rats. Methods: Thirty-six male Wistar rats were fed either a normal diet or an HFD for 24 weeks. At week 13, HFD-fed rats received either 50, 100, and 500 mg/kg/day of PSO or 300 mg/kg/day metformin for 12 weeks. After 24 weeks, the metabolic parameters, gut microbiota, gut barrier, inflammation, and oxidative stress were determined. Results: HFD-fed rats showed gut dysbiosis, gut barrier disruption with inflammation, increased oxidative stress, metabolic endotoxemia, and insulin resistance. Treatment with PSO and metformin not only effectively attenuated gut dysbiosis, but also improved gut barrier integrity and decreased gut inflammation. PSO also decreased oxidative stress, metabolic endotoxemia, and insulin resistance in HFD-fed rats. Metformin had greater benefits than PSO. Conclusion: PSO and metformin had the beneficial effect on attenuating gut inflammation and metabolic disturbance in obese-insulin resistance.

## 1. Introduction

Obesity is considered a major public health issue worldwide that is closely associated with the development of several chronic complications, such as metabolic syndrome, diabetes, cardiovascular diseases, inflammatory bowel diseases, and neurodegenerative diseases [1]. Obesity is caused by the interaction of genetic, behavioral, and environmental factors [2]. The most common cause of obesity is overeating due to a high-caloric diet or maintaining a poor diet. Recent investigations have reported that a prolonged high-fat diet (HFD) consumption induced intestinal inflammatory responses, leading to the development of obesity and insulin resistance [3,4,5]. Chronic low-grade inflammation with a disturbance of gut microbiota, called “gut dysbiosis”, has been observed in obese conditions in mice [6,7]. Gut microbiota positively influences host health by boosting host-immune responses, improving digestive function, regulating energy metabolism, and protecting the host against pathogen colonization [8]. Our previous studies have shown that rats with chronic HFD ingestion developed not only obesity but also gut dysbiosis, gut inflammation, systemic inflammation, and metabolic disturbances, including insulin resistance and dyslipidemia [9,10]. HFD-induced gut dysbiosis led to the growth of several harmful bacteria, particularly bacteria producing lipopolysaccharides (LPS), resulting in an increase in gut permeability, as indicated by downregulated expression of intestinal tight junctions and destroyed intestinal mucus layers [11]. LPS is subsequently translocated from the gut into the systemic circulation, triggering inflammatory responses to several tissues, such as the liver and adipose tissue. A high concentration of LPS in the systemic circulation, also known as metabolic endotoxemia, was found in the pathophysiology of obesity and insulin resistance [7].

*Perilla frutescens* (Nga-Mon) is a member of the mint family, *Lamiaceae*. It has been commonly used as a functional food and traditional medicine in Asian countries, including China, Japan, Korea, India, and northern Thailand [12]. Perilla seed oil (PSO) is a good source of omega-3 unsaturated fatty acids (PUFAs), especially alpha-linolenic acid (ALA) and had several health benefits with anti-oxidant, anti-inflammation, anti-obesity, cardioprotective, and neuroprotective properties [12]. Our previous study found that the proportion of omega-3 to omega-6 PUFA in a cold-pressed extraction of PSO was 4:1 [13]. Dietary supplementation with a high ratio of omega-3 to omega-6 PUFA has been found to improve pathological conditions in obesity, including systemic inflammation and insulin resistance, by suppressing the activation of the toll-like receptors 4 (TLR4) signaling pathway [14]. ALA is abundant in oil extracted from the seed of perilla, sacha inchi, rapeseed, and linseed. Several studies have found that ALA exerted anti-inflammatory agents, resulting in the prevention of some chronic diseases [15,16,17]. For the beneficial effects of PSO, five studies in an HFD-fed model and two studies in a diabetes model were used to investigate the alterations of metabolic and gut function following PSO treatment [18,19,20,21]. Dietary supplementation of PSO not only increased ALA, eicosapentanoic, and docosahexanoic acid, but also shift the composition of the gut microbiota and modulated villus morphology in normal mice [22]. In HFD-fed mice, PSO protected metabolic dysfunction and inflammation by inhibiting myeloid differentiation 88 (MyD88) of TLR4 signaling in adipose tissue [18]. PSO also declined lipid accumulation in aortic and hepatic induced by HFD through regulating lipogenesis and lipolysis [16]. Recent studies demonstrated that PSO-rich diet supplementation in rats for 16 weeks alleviated HFD-induced hepatic steatosis, inflammation, and gut dysbiosis by enhancing the abundance of *Prevotella* and *Escherichia* and decreased endoplasmic reticulum (ER) stress-mediated autophagy [17,20]. In addition, PSO attenuated the severity of colitis in HFD-fed mice, as indicated by decreasing inflammatory mediators, such as inducible nitric oxide and cyclooxygenase 2, and inhibited nuclear factor-kappa B activation (NF-κB) activation in the colon [19]. In type-two diabetic KKAy mouse model, supplementation of PSO regulated gut microbiota, improved hypertriglyceridemia, and ameliorated insulin resistance by enhancing the liver expression of phosphoinositide-3 kinase (PI3K) and protein kinase B (AKT) pathways [15,21]. However, the effect of PSO rich in omega-3 PUFAs on gut microbiota, gut inflammation, and metabolic disturbances in HFD-induced obese-insulin-resistant conditions in rats has not been well defined. Furthermore, metformin is the first line of the drug to treat obese-insulin-resistant diseases, and it has been shown to improve gut inflammation and metabolic dysfunction in obese-insulin resistance [23,24]. Therefore, this study aimed to compare the effects of different doses of PSO and metformin on gut microbiota, gut inflammation, intestinal barrier integrity, systemic inflammation, and metabolic parameters induced by HFD consumption in obese-insulin-resistant rats.

## 2. Materials and Methods

### 2.1. Preparation of Perilla Seed Oil and Analysis of the Composition of Fatty Acid (FA) 

Perilla seeds were collected from major cultivation regions in the Phayao province. The oil was extracted using a cold-pressed extractor. The FA compositions of the PSO were then measured through an Agilent 6890N Gas Chromatography-Mass Spectrometer (GC-MS) system (Agilent Technologies, Wilmington, DE, USA) by using Central Laboratory (Thailand) Company Limited in accordance with the in-house protocol based on the AOAC method 996.06 (AOAC, revised 2001). The FA content in the PSO was presented as a percentage of the total FAs. The main FAs of the PSO were ALA (59.20 ± 0.11%), followed by linoleic acid (LA, 17.98 ± 0.34%), oleic acid (OA, 11.93 ± 0.31%), palmitic acid (PA, 7.82 ± 0.55%), stearic acid (SA 3.01 ± 0.04%), and other fatty acids (0.58 ± 0.13%).

### 2.2. Animals

**Figure 1:**
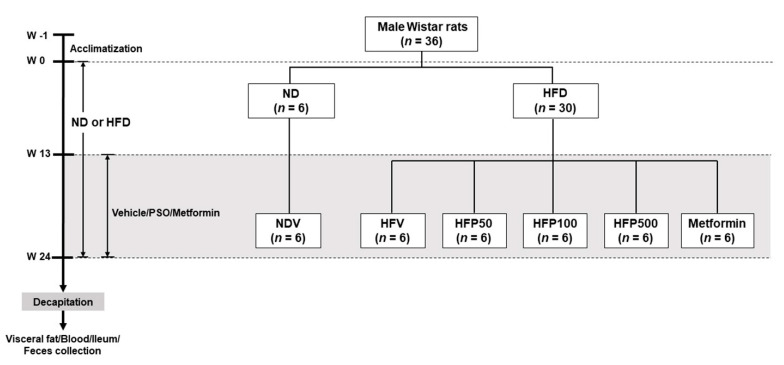
Schematic representation of the animal experimental design in this study. NDV = Normal diet-fed rats treated with the vehicle; HFV = High fat diet-fed rats treated with vehicle; HFP50 = HFD-fed rats treated with 50 mg/kg of PSO; HFP100 = HFD-fed rats treated with 100 mg/kg of PSO; HFP500 = HFD-fed rats treated with 500 mg/kg of PSO; HFM = HFD-fed rats treated with 300 mg/kg of metformin.

A total of 36 male Wistar rats (initial weights 180–200 g) were procured from Nomura Siam International, Bangkok, Thailand. All animal experiments and procedures were approved by the Ethics Committee of the Laboratory Animal Center, Chiang Mai University, Thailand (approval No. RT005/2562[02/2562-04-18] on 21 May 2019). All rats were housed in a plastic cage (two per cage) in a room with a controlled temperature (25 ± 1 °C), lighting (12 h light-dark cycle), and relative humidity. After a one-week acclimatization period, the rats were randomly assigned into two dietary groups and given either a normal diet (ND; 19.77% energy from fat, *n* = 6) or a high-fat diet (HFD; 59.28% energy from fat, *n* = 30) for 24 weeks [25]. The food intake and body weight of all rats were observed weekly. At week 13, ND-fed rats were treated with deionized water as a vehicle for an additional 12 weeks (NDV; *n* = 6). HFD-fed rats were separated into five subgroups (*n* = 6 for each subgroup). Each subgroup was orally gavaged with either vehicle (corn oil; HFV), 50 mg/kg perilla seed oil (PSO; HFP50), 100 mg/kg PSO (HFP100), 500 mg/kg PSO (HFP500), or 300 mg/kg metformin [26] (Novartis’ Galvus, Bangkok, Thailand; HFM) for an additional 12 weeks. The PSO was dissolved in a vehicle of corn oil because it lacked ALA. Metformin was used as a reference drug and dissolved in sterile drinking water. Following a 24-week experimental period, the rats were food-deprived for a minimum of 5 h. After the rats were anesthetized with isoflurane, plasma was collected from the tail vein to determine the glucose, insulin, lipid profiles, malondialdehyde (MDA), and LPS levels. The ileal and fecal samples were collected, immediately placed in liquid nitrogen, and stored at −80 °C until analysis. All experimental designs are presented in Figure 1.

### 2.3. Measurements of Metabolic Parameters

Plasma glucose, total cholesterol (TC), and triglyceride (TG) levels were measured using a colorimetric assay kit (ERBA Mannheim, Mannheim, Germany). Plasma insulin level was determined using the sandwich enzyme-linked immunosorbent assay kit (Millipore, MI, USA). Plasma high-density lipoprotein (HDL) level was measured using a colorimetric assay kit (Biovision Inc., Milpitas, CA, USA). Plasma low-density lipoprotein (LDL) was estimated from Friedewald’s equation [27]. The degree of insulin resistance was assessed using the Homeostasis Model Assessment (HOMA) index, which was calculated by [insulin (μU/mL) × glucose (mmol/L)]/22.5. A higher HOMA index was interpreted as a higher degree of insulin resistance [28,29].

### 2.4. Fecal Microbiota Analysis

The fecal samples of four rats in each group were collected and stored at −80 °C. Genomic DNA was extracted from the frozen fecal samples (~250 mg) using a QIAamp PowerFecal Pro DNA kit (QIAGEN, Germany) and following the protocol’s instructions. The population of the gut microbiota (*Firmicutes*, *Bacteroidetes*, and *Enterobacteriaceae*) was analyzed using qPCR. As described in our previous study, the DNA was then subjected to qPCR using a SensiFAST SYBR Lo-ROX kit (Bioline, Taunton, MA, USA) with the bacterial plasmids [10]. The number of gene copies in each bacterial population was analyzed according to the standard curves that were produced from bacterial 16S rRNA gene fragments, including *Eubacteria* (*R. productus*), *Firmicutes/Clostridiales* (*R. productus*), *Firmicutes/Lactobacillales* (*L. acidophilus*), *Bacteroidetes* (*B. fragilis*), and *Enterobacteriaceae* (*E. coli* TOP10) [10,30]. The results were expressed as the percentage of each bacterial in the *phylum* level divided by the *Eubacteria* level.

### 2.5. Determination of Periodic Acid-Schiff Staining

Periodic Acid-Schiff (PAS) staining was determined to visualize the mucus-secreting goblet cells. Distal ileal tissue from each rat was collected, washed in PBS, and fixed in 10% neutral-buffered formalin for 24 h. The specimens subsequently were embedded in paraffin wax and sectioned at five-μm thickness, respectively. The paraffin slides were stained using Schiff’s Reagent for 10 min. The stained sections were examined and photographed under a light microscope at ×400 magnification.

### 2.6. Western Blot Analysis

Ileal tissue was homogenized in a lysis buffer to extract proteins. Total protein (60 g) was subjected to western blotting by separation using 10% SDS polyacrylamide gel electrophoresis and transferring to a nitrocellulose membrane in a transfer system (Bio-Rad Laboratories, Hercules, CA, USA). The membrane was blocked in 5% bovine serum albumin (BSA) in TBS-T buffer (20 mM Tris–HCl (pH 7.6), 137 mM NaCl, and 0.05% Tween). The membranes were then incubated with anti-ZO-1 and anti-actin (Santa Cruz, Delaware, CA, USA) antibodies overnight at 4 °C. Secondary antibodies (Cell Signaling Technology, Danvers, MA, USA) were incubated for 1 h at room temperature. Protein expression was visualized using an enhanced chemiluminescence detection kit (Bio-Rad Laboratories, CA, USA).

### 2.7. Reverse Transcription-Quantitative Polymerase Chain Reaction (RT-qPCR)

The expression of genes related to inflammatory factors in the ileum, including TNF-α and IL-1β, was detected using RT-qPCR. Briefly, ileal tissues were kept in RNAlater™ Stabilization Solution (Thermo Fisher Scientific, Waltham, MA, USA). Total ileal RNA was isolated using TRIzol reagent following the supplier’s protocol (Ambion, Life Technologies, Foster City, CA, USA). The cDNA was generated from 2 μg of total RNA using the Tetro cDNA synthesis kit (Bioline, Taunton, MA, USA). The qPCR was performed by using SensiFAST SYBR^®^ Lo-ROX Kit (Bioline, London, UK). The relative gene expressions for TNF-α and IL-1β were normalized to the internal control actin and analyzed using the 2^−∆∆CT^ procedure. The PCR primers in this study were as follow: rat TNF-α primer 5′-AAATGGGCTCCCTCTCATCAGTCC-3′ (forward), 5′-TCTGCTTGGTGGTTTGCTACGAC-3′ (reverse); rat IL-1β primer 5′-CACCTCTCAAGCAGAGCACAG-3′ (forward), 5′-GGGTTCCATGGTGAAGTCAAC-3′ (reverse); and rat actin primer 5′-GACATGCCGCCTGGAGAAAC-3′ (forward), 5′-AGCCCAGGATGCCCTTTAGT-3′ (reverse).

### 2.8. Determination of the Serum Lipopolysaccharide Level

The serum lipopolysaccharide (LPS) level in the rat was determined using the Pierce Limulus Amoebocyte Lysate (LAL) Chromogenic Endotoxin Quantitation Kit (Thermo Fisher Scientific, Rockford, IL, USA), according to the protocol’s instructions.

### 2.9. Determination of the Tissue and Serum MDA

The MDA level was used as an oxidative stress marker and was determined using an HPLC assay. Briefly, homogenate tissue or serum was mixed with 10% trichloroacetic acid containing BHT (50 ppm). The sample was incubated in a water bath at 90 °C for 30 min and then centrifuged at 6000 rpm for 10 min. The clear supernatant was transferred into a new tube, 0.44 M H_3_PO_4_ plus 0.6% thiobarbituric acid was added and it was incubated at 90 °C for 30 min. The pink solution of thiobarbituric acid reactive substances (TBARS) was filtered using a polysulfone membrane. The ileal tissue and serum MDA concentration were represented in μmol/g protein and μM, respectively.

### 2.10. Statistical Analysis

Data are expressed as the means ± standard error mean (SEM). Statistical comparisons were analyzed using a one-way ANOVA followed by Fisher’s least significance difference (LSD) analysis *post hoc* analysis for testing the difference between groups. Statistical significance was assumed at a *p*-value < 0.05.

## 3. Results

### 3.1. Perilla Seed Oil Attenuated Peripheral Insulin Resistance and Hyperlipidemia in Obese-Insulin-Resistant Rats

Body weight and visceral fat weight were significantly more elevated in the HFD-fed rats treated with vehicle (HFV) than they were in the ND-fed rats treated with the vehicle (NDV) (Table 1). Metformin, but not all doses of perilla seed oil (PSO) treatment, significantly reduced the gain of body weight and visceral fat weight in the HFD-fed rats (Table 1). Treatments with 100 and 500 mg/kg/day of PSO and metformin markedly diminished the plasma insulin level and HOMA index without changing the plasma glucose level when compared to the HFV group (Table 1). The HFV group showed hyperlipidemia, as demonstrated by increased plasma TC, TG, and LDL levels (Table 1). Treatments of 100 and 500 mg/kg/day of PSO and metformin significantly decreased those parameters in HFD-fed rats (Table 1). The only treatment of metformin has significantly raised the HDL levels in HFD-fed rats (Table 1). These results revealed that 100 and 500 mg/kg/day of PSO and metformin ameliorated metabolic disturbance in HFD-fed rats. Surprisingly, the effect of PSO on the metabolic parameters was in a dose-independent manner.

### 3.2. Perilla Seed Oil Attenuated Gut Dysbiosis in Obese-Insulin-Resistant Rats

**Figure 2:**
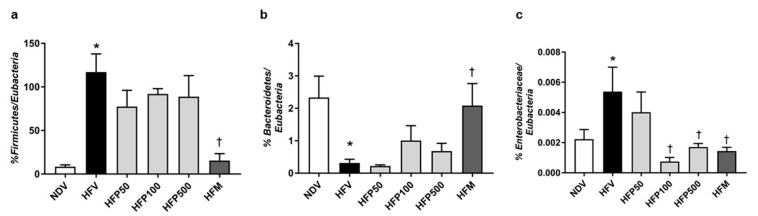
The effect of PSO administration on the composition of the gut microbiota in HFD-fed rats. (**a**) Percentage of *Firmicutes/Eubacteria*; (**b**) Percentage of *Bacteroidetes/Eubacteria*; (**c**) Percentage of *Enterobacteriaceae/Eubacteria*. Data are presented as mean ± SEM, * *p* < 0.05 versus the NDV group; ^†^ *p* < 0.05 versus the HFV group. HFD = High fat diet; NDV = Normal diet-fed rats treated with the vehicle; HFV = High fat diet-fed rats treated with vehicle.

The effect of PSO on gut microbiota at the *phylum* level was performed using 16S rRNA targeted-qPCR analysis. HFV rats demonstrated gut dysbiosis by a significant increase in the percentage of *Fimicutes/Eubacteria* (Figure 2a), a decline in the percentage of *Bacteroidetes/Eubacteria* (Figure 2b), and an increase in the percentage of *Enterobacteriaceae/Eubacteria* (Figure 2c) in comparison to NDV rats. Metformin, but not all doses of PSO, reversed gut dysbiosis, as displayed by a significant reduction in the percentage of *Fimicutes/Eubacteria* and an increase in the percentage of *Bacteroidetes*/*Eubacteria* in HFD-fed rats (Figure 2a,b). Interestingly, treatments with 100 and 500 mg/kg/day of PSO and metformin equally reduced the percentage of *Enterobacteriaceae/Eubacteria* in HFD-fed rats (Figure 2c). Although 100 and 500 mg/kg/day of PSO attenuated gut dysbiosis, particularly the percentage of *Enterobacteriaceae*, in HFD-fed rats in a dose-independent manner, metformin had the highest efficacy in alleviating gut dysbiosis.

### 3.3. Perilla Seed Oil Improved Intestinal Barrier Integrity in Obese-Insulin-Resistant Rats

**Figure 3:**
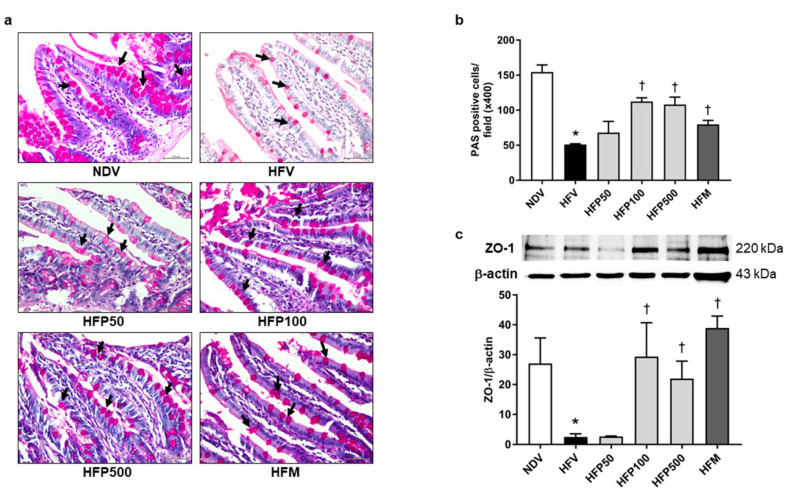
Effect of PSO on intestinal barrier integrity in obesity-induced insulin-resistant rats. (**a**) Representative image of PAS-positive goblet cells indicated by black arrows, ×400 magnification; (**b**) The percentage of goblet-positive cells was analyzed using light microscopy with a high-power field (×400 magnification) from at least five fields; (**c**) Expression of ZO-1 in the ileum. Data are presented as mean ± SEM, * *p* < 0.05 versus the NDV group; ^†^ *p* < 0.05 versus the HFV group. PSO = Perilla seed oil; PAS = Periodic Acid-Schiff; ZO-1 = Zonula occludens-1; NDV = Normal diet-fed rats treated with the vehicle; HFV = High fat diet-fed rats treated with vehicle.

In the current study, intestinal barrier integrity was determined by the goblet cell quantification and tight junction protein levels in the ileum. Goblet cell quantification and tight junction protein levels were determined by PAS staining and ZO-1 expression, respectively. The HFV group significantly decreased intestinal barrier integrity, as indicated by decreased PAS-positive cells (Figure 3a,b), and downregulated the expression of tight junction ZO-1 in the ileum (Figure 3c) in comparison to the NDV group. Interestingly, treatment with 100 and 500 mg/kg/day of PSO and metformin equally increased PAS-positive cells (Figure 3a,b) and tight junction ZO-1 expression (Figure 3c) in comparison to the HFV group. These results showed that 100 and 500 mg/kg/day of PSO and metformin equally ameliorated the disruption of intestinal barrier integrity in HFD-fed rats.

### 3.4. Perilla Seed Oil Reduced Intestinal Inflammation and Oxidative Stress Level in Obese-Insulin-Resistant Rats

**Figure 4:**
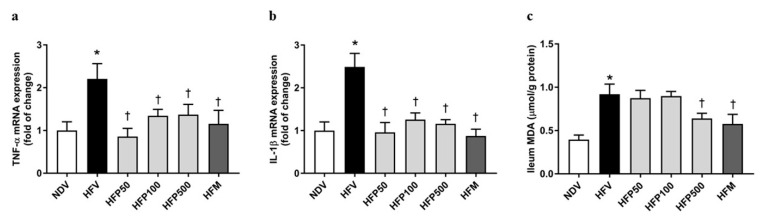
Effect of PSO on intestinal inflammation in obesity-induced insulin-resistant rats. (**a**) TNF-α mRNA expressions; (**b**) IL-1β mRNA expressions; (**c**) MDA levels. Data are presented as mean ± SEM, * *p* < 0.05 versus the NDV group; ^†^ *p* < 0.05 versus the HFV group. PSO = Perilla seed oil; TNF-α = Tumor necrosis factor-alpha; IL-1β = Interleukin 1 beta; MDA = Malondialdehyde; NDV = Normal diet-fed rats treated with the vehicle; HFV = High fat diet-fed rats treated with vehicle.

In the HFV group, the expression of inflammatory factor genes in the ileum, including TNF-α (Figure 4a) and IL-1β (Figure 4b), significantly increased in comparison to that of the NDV group. Interestingly, treatment with all doses of PSO and metformin equally decreased the ileal expression of TNF-α (Figure 4a) and IL-1β (Figure 4b) in HFD-fed rats, suggesting that all doses of PSO and metformin equally ameliorated the intestinal inflammation induced by HFD in rats.

The MDA in the ileum was used for the assessment of oxidative stress generation, which was caused by the disruption of the gut barrier integrity, and consequently, augmented gut permeability and stimulated gut inflammatory responses [31]. We found that the HFV group showed a more significant increase in the intestinal oxidative stress level in the ileum, as indicated by the increased MDA level, than the NDV group (Figure 4c). Treatment with 500 mg/kg/day of PSO and metformin remarkably reduced the ileal MDA level of HFD-fed rats (Figure 4c). These findings revealed that only 500 mg/kg/day of PSO and metformin ameliorated intestinal oxidative stress in rats who received HFD feed.

### 3.5. Perilla Seed Oil Attenuated Systemic Inflammation and Oxidative Stress in Obese Insulin-Resistant Rats

**Figure 5:**
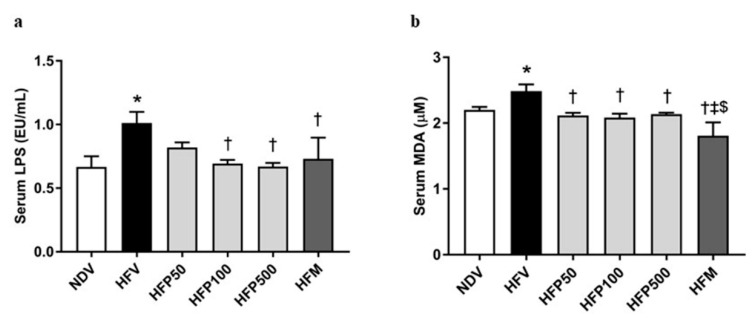
The effect of PSO on endotoxemia and oxidative stress in obesity-induced insulin-resistant rats. (**a**) Serum LPS level; (**b**) Serum MDA level. Data are presented as mean ± SEM, * *p* < 0.05 versus the NDV group; ^†^ *p* < 0.05 versus the HFV group; ^‡^ *p* < 0.05 versus HFP50; ^$^ *p* < 0.05 versus HFP500.

Metabolic endotoxemia is indicated by an elevated plasma LPS, which is produced from gram-negative bacteria, and is linked to HFD-induced systemic inflammatory response [32]. The HFV group significantly increased systemic inflammation, as demonstrated by a greater increase in serum LPS levels (Figure 5a) than in the NDV group. Interestingly, treatment with 100 and 500 mg/kg/day of PSO and metformin equally reduced serum LPS levels in HFD-fed rats (Figure 5a). Our results indicated that 100 and 500 mg/kg/day of PSO and metformin attenuated LPS generation from gram-negative bacteria in HFD-fed rats.

The HFV group also showed significantly increased systemic oxidative stress, as displayed by increased serum MDA levels (Figure 5b), compared to that of the NDV group. Interestingly, all doses of PSO and metformin significantly diminished the serum MDA level in HFD-fed rats (Figure 5b). In addition, metformin was more effective than PSO in reducing systemic oxidative stress levels in HFD-fed rats (Figure 5b). Our results indicated that all doses of PSO and metformin ameliorated both systemic oxidative stress in HFD-fed rats, but that metformin had the highest efficacy.

## 4. Discussion

The significant findings of the present study are as follows: (1) an HFD intake for 24 weeks caused gut dysbiosis, intestinal barrier integrity disruption, intestinal inflammation, systemic inflammation, peripheral insulin resistance, and hyperlipidemia in rats; (2) PSO treatment, specifically, 100 and 500 mg/kg/day of PSO and metformin, equally attenuated HFD-induced intestinal barrier dysfunction, intestinal inflammation, systemic inflammation, peripheral insulin resistance, and hyperlipidemia; and (3) metformin treatment for HFD-fed rats better-alleviated gut dysbiosis than PSO, resulting in greater improvement in intestinal and systemic oxidative stress levels than PSO with PSO treatment.

The intestinal microbiota has been found to play an essential role in various physiological functions in the host [33]. Increasing evidence has shown that a disturbance of gut microbiota composition, as indicated by gut dysbiosis, is one of the hallmarks of the pathogenesis of chronic inflammatory diseases, such as cardiovascular disease, metabolic syndrome, obesity, diabetes mellitus, and inflammatory bowel disease in animals and humans [2,34]. The chronic consumption of HFD is one of the main causes of gut dysbiosis, causing, in particular, increased gram-negative bacteria resulting in elevated LPS levels in plasma [35,36,37]. In our study, a 24-week HFD consumption altered gut microbiota composition by increasing *Firmicutes* while decreasing *Bacteroidetes*. In addition, *Enterobacteriaceae* bacteria containing LPS were also increased, accompanied by increasing LPS plasma levels in HFD-fed rats. The excessive bacterial LPS activated immune responses, in turn activating the inflammatory responses and generating oxidative stress in the intestinal mucosa [11,38]. A previous study showed that these changes damaged the mucosal intestinal barrier, which resulted in intestinal inflammation, systemic inflammation, and metabolic disturbances [39]. Our results similarly showed that the long-term ingestion of HFD induced gut barrier impairment, intestinal inflammation, systemic inflammation, hyperlipidemia, and insulin resistance. Thus, we proposed that increased intestinal dysbiosis in HFD-fed rats caused intestinal barrier integrity disruption, leading to increased gut permeability, intestinal inflammation, hyperlipidemia, and peripheral insulin resistance. Similar to our previous study, all these mechanisms caused metabolic disturbance induced by gut dysbiosis in HFD-fed rats [37].

In the present study, we showed that PSO, which contains high amounts of PUFA, attenuated gut dysbiosis, intestinal barrier integrity disruption, intestinal inflammation, intestinal oxidative stress, systemic inflammation, and oxidative stress, and also decreased dyslipidemia and peripheral insulin resistance in HFD-fed rats. Similarly, long-term dietary supplementation with PSO ameliorated gut dysbiosis in HFD-induced colon inflammation by reducing the number of *Enterobacteriaceae* and elevating the number of *Bifidobacteria*, resulting in an increase in tight junction expression and a reduction in pro-inflammatory cytokine production [40]. Dietary supplementation with PSO for 16 weeks has shown lessened the HFD-induced nonalcoholic fatty liver disease and hepatic inflammation in an animal model [17]. A previous study demonstrated that a mixture of fish oil and krill oil, ALA-rich oil, modulated gut microbiota and decreased HFD-induced obesity in mice [41]. Notably, the administration of *Enterobacter*, belonging to the phylum *Proteobacteria*, in germ-free mice showed increased serum LPS and activated inflammatory conditions, leading to induced obesity and insulin resistance [42]. Thus, LPS plasma has been used in accordance with changing gut microbiota and increasing gut permeability [32]. Our data consistently demonstrated that a high dose of PSO treatment reduces the abundance of *Enterobacteriaceae*, but cannot alter the compositions of *Firmicutes* and *Bacteroidetes* in HFD-fed rats, leading to diminished LPS levels in plasma. These results suggest that the existence of the dose-independent effects of PSO treatment against HFD-induced insulin resistance may partially result from altered strain specific gut microbiota.

Our study additionally showed that 100 and 500 mg/kg/day of PSO treatment prevented intestinal barrier destruction in HFD-fed rats by protecting mucus-secreting goblet cells and upregulating the tight junction ZO-1 proteins in the ileum. All doses of PSO also attenuated the inflammatory responses and oxidative stress levels of the ileum in the HFD-fed rats by reducing the expression of pro-inflammatory cytokines (TNF-α and IL-1β) and MDA levels. A recent study reported that PSO alleviated HFD-induced colonic inflammation by suppressing the NF-κB pathway along with upregulating tight junction ZO-1 expression [18]. Our findings and those of others indicate that PSO exhibits an anti-inflammatory property against HFD-induced intestinal barrier destruction in obese-insulin-resistant rats.

HFD consumption induced an increase in plasma LPS levels, also known as metabolic endotoxemia, which is associated with systemic inflammation, resulting in the development of metabolic disorders [39]. Our study showed that the treatment of PSO in doses of 100 and 500 mg/kg/day reduced LPS and MDA levels in plasma, leading to reduced systemic inflammation, peripheral insulin resistance, and hyperlipidemia in a dose-independent manner. Although PSO decreased peripheral insulin resistance, it did not reduce body weight gain and visceral fat weight in HFD-fed rats. The gut microbiota is associated with energy harvest, resulting from *Firmicutes* being more capable of extracting energy from food than *Bacteroidetes*, thus eventually increasing the absorption of calories and weight gain [43]. According to this finding, PSO treatment did not affect the *Firmicutes* levels in HFD-fed rats and thereby did not prevent the body weight gain and vesical fat weight in HFD-fed rats. Surprisingly, the 100 and 500 mg/kg/day of PSO treatment significantly reduced dyslipidemia in HFD-fed rats, as displayed by the lowered TC, TG, and LDL levels but unaffected HDL levels. Other studies have shown that supplementation with PSO ameliorated HFD-induced dyslipidemia by suppressing lipogenesis and increasing lipid oxidation in the liver [17,40]. PSO also decreased serum cholesterol, and arterial and hepatic lipid aggregation by regulating lipogenesis and lipolysis pathways in HFD mice [16]. Therefore, we speculated that PSO may suppress lipogenesis and increase lipolysis in the liver of HFD-fed rats, resulting in reduced dyslipidemia.

Our study is the first to compare the efficacy of different doses of PSO and metformin treatment in obese-insulin-resistant rats. Metformin is a commonly used medicine to treat type 2 diabetes mellitus. Metformin exerts a hypoglycemic effect by effectively controlling the blood glucose of obese and diabetic patients [44]. Furthermore, metformin modulates the gut microbiota, leading to maintaining gut barrier integrity, enhancing the short-chain fatty acid production, and improving glucose homeostasis [45,46]. Metformin has evidenced that improving the expression of tight junction occludin-1 in the gut increased the abundance of beneficial bacteria, such as *Lactobacillus* and *Akkermansia muciniphila*, attenuated endotoxemia and oxidative stress, and promoted the anti-oxidative Nrf2 system, resulting in an improved glucose metabolism and insulin signaling pathway in the liver and the muscles of HFD-fed mice [47]. Interaction between metformin and gut microbiota has shown both therapeutic and adverse effects [48,49]. The beneficial effect of metformin on improving glycemic control and anti-inflammatory properties in type 2 diabetes patients was modulated by intestinal microbiota composition through the modulation of mucin-degrading *A. muciniphila* and short-chain fatty acid (SCFA)-producing microbiota [48]. However, metformin led to a shift of the gut microbiota, which mediated mechanisms of intestinal adverse effects by increasing the abundance of *Escherichia* species [49,50]. Interestingly, our results showed that metformin treatment at a dose of 300 mg/kg was more beneficial than PSO treatment, as evidenced by the markedly improved gut dysbiosis and systemic inflammation. Thus, metformin may regulate the anti-oxidant status and systemic inflammation through modulated intestinal microbiota, which could contribute to prevention in obese-insulin-resistant rats. The prolonged use of metformin, however, has demonstrated severe adverse effects, including lactic acidosis [51]. This study indicates that the treatment of 100 mg/kg/day of PSO is similar but slightly less effective than metformin in attenuating obese-insulin-resistant conditions.

For the clinical studies, the treatment of two capsules of PSO (500 mg/capsule), four times daily for six months, was safe in dementia patients [52]. In addition, the consumption of 7.0 mL/day of PSO for 12 months increased ALA and EPA in blood levels associated with enhancing mental health in Japanese healthy adults [53]. However, there was only one study that determined the effect of PSO on metabolic function. That study demonstrated that treatment with PSO decreased dyslipidemia and inflammatory biomarkers without adverse effects in patients with hyperlipidemia [54]. Furthermore, the effect of PSO on intestinal microbiota and function in an obese patient has never been investigated. Therefore, further studies are required to provide more clinical evidence to strengthen the effect of PSO on the alteration of metabolic and gut functions in obese people.

## 5. Conclusions

**Figure 6:**
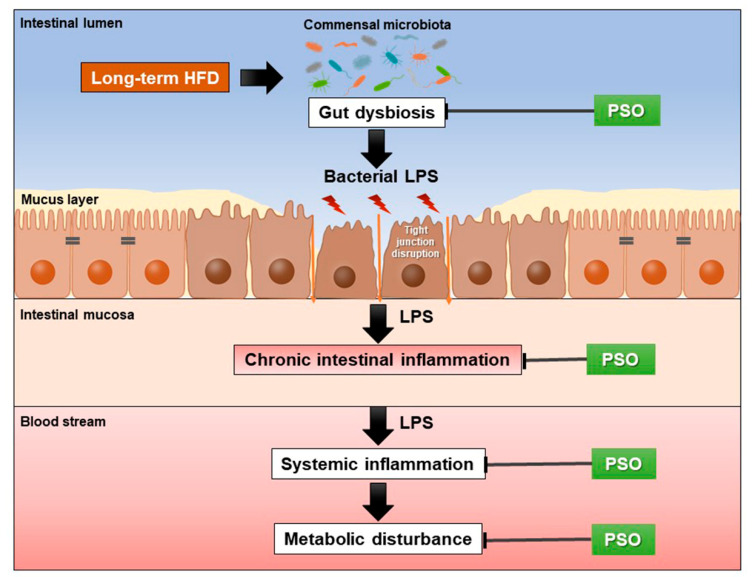
This schematic overview shows the effects of PSO treatment on obese-insulin-resistant rats. PSO alleviated HFD-induced gut dysbiosis, which results in decreased intestinal inflammation, systemic inflammation, and metabolic disturbance. HFD = high fat diet; LPS = lipopolysaccharide; PSO = perilla seed oil.

Treatment with either PSO or metformin attenuated HFD-induced gut dysbiosis, intestinal inflammation, and metabolic disturbance. We proposed that PSO decreased gut dysbiosis in HFD-fed rats, and consequently, reduced intestinal barrier integrity disruption and intestinal inflammation. These changes lessened systemic inflammation and oxidative stress, leading to reduced dyslipidemia and peripheral insulin resistance in PSO-treated HFD-fed rats. The proposed mechanism of the effect of PSO treatment on the gut and metabolic functions in obese-insulin-resistant conditions is represented in Figure 6. Thus, PSO could be further developed as a potential functional food and considered for preventing obesity-induced insulin resistance when metformin use is discontinued.

## Figures and Tables

**Table 1:** Effects of PSO and metformin treatment on metabolic parameters in rats.

Parameters	Groups					
	NDV	HFV	HFP50	HFP100	HFP500	HFM
Body weight (g)	525.5 ± 8.27	670.8 ± 27.09 *	672.2 ± 24.95 *	659.2 ± 8.67 *	698.7 ± 17.67 *	605.5 ± 19.36 *^,†,‡,#,$^
Visceral fat (g)	27.21 ± 2.60	61.89 ± 4.45 *	65.36 ± 3.63 *	63.79 ± 4.68 *	69.1 ± 4.21 *	48.42 ± 5.34 *^,†,‡,#,$^
Fasting glucose (mg/dL)	135.1 ± 3.43	144.9 ± 13.55	137.8 ± 4.13	133.5 ± 5.79	133.3 ± 5.5	140.3 ± 8.99
Fasting insulin (ng/mL)	7.82 ± 0.55	13.10 ± 1.99 *	8.96 ± 0.31 ^†^	8.94 ± 1.11 ^†^	8.83 ± 0.89 ^†^	8.59 ± 0.57 ^†^
HOMA index	63.04 ± 5.92	120.1 ± 32.52 *	85.89 ± 5.59	69.83 ± 10.89 ^†^	72.12 ± 6.75 ^†^	63.35 ± 9.40 ^†^
Total cholesterol (mg/dL)	113.8 ± 5.71	151.7 ± 18.44 *	131.2± 7.02	100.7 ± 14.64 ^†^	117.4 ± 7.72 ^†^	103.4 ± 7.11 ^†^
HDL (mg/dL)	39.96 ± 2.42	29.96 ± 3.35 *	33.29 ± 1.42	35.44 ± 1.99	33.57 ± 1.93	40.35 ± 1.56 ^†^
LDL (mg/dL)	26.55 ± 7.56	90.92 ± 17.07 *	81.09 ± 6.97	53.42 ± 8.09 ^†^	60.9 ± 4.58 ^†^	46.27 ± 10.81 ^†^
Triglyceride (mg/dL)	114.1 ± 7.51	161.6 ± 9.07 *	107.1 ± 7.86 ^†^	107.3 ± 21.68 ^†^	108.4 ± 7.38 ^†^	99.5 ± 6.61 ^†^

Data are presented as mean ± SEM. * *p* < 0.05 versus NDV; ^†^ *p* < 0.05 versus HFV; ^‡^ *p* < 0.05 versus HFP50; ^#^ *p* < 0.05 versus HFP100; ^$^ *p* < 0.05 versus HFP500; *n* = 6 rats/group; NDV = Normal diet-fed rats treated with the vehicle; HFV = High fat diet-fed rats treated with vehicle; HFP50 = HFD-fed rats treated with 50 mg/kg of PSO; HFP100 = HFD-fed rats treated with 100 mg/kg of PSO; HFP500 = HFD-fed rats treated with 500 mg/kg of PSO; HFM = HFD-fed rats treated with 300 mg/kg of metformin; HOMA = Homeostasis Model Assessment; HDL = High-density lipoprotein; LDL = Low-density lipoprotein.

## Data Availability

The data presented in this study are available on request from the corresponding author.
